# Assessment of the relationship between plasma fibrinogen-to-albumin ratio and slow coronary flow phenomenon in patients without obstructive coronary artery disease

**DOI:** 10.1186/s12872-023-03579-z

**Published:** 2023-11-06

**Authors:** Shao-bing Yang, Ying Cui, Jian-jun Hou, Hui Zhang, Xiao-yang Pei, Yong Wang

**Affiliations:** 1 Department of Cardiology, The General Hospital of Ningxia Medical University, Yinchuan, China; 2 Department of Cardiology, Shenzhen Luohu Hospital Group Luohu People’s Hospital (The Third Affiliated Hospital of Shenzhen University), Shenzhen, China

**Keywords:** Fibrinogen to plasma albumin ratio, Slow coronary flow fhenomenon, Predictors

## Abstract

**Background:**

Prior studies have suggested that the chronic inflammatory response has an important role in the pathophysiology of slow coronary flow phenomenon (SCFP). However, data are scarce regarding the role of plasma fibrinogen-to-albumin ratio (PFAR) in patients having SCFP without obstructive coronary artery disease (CAD). In this study, we investigated the relationship between PFAR and the presence of SCFP in patients without obstructive CAD.

**Methods:**

From January 2021 to January 2023, we consecutively recruited 1085 patients without obstructive CAD according to the diagnostic and exclusion criteria. In total, SCFP was diagnosed in 70 patients. A 1:2 age-matched case–control study was then conducted using comparators without SCFP. Ultimately, this study enrolled 70 patients with angiographically normal coronary arteries and SCFP, along with 140 comparators with angiographically normal coronary arteries and normal coronary flow. Plasma fibrinogen and albumin levels were measured, and the PFAR was then calculated for each patient.

**Results:**

PFARs were significantly greater in the SCFP group than in the comparators with normal coronary flow (82.8 ± 15.4 vs 73.1 ± 19.5, *p* < 0.001). PFAR increased with increasing numbers of vessels affected by SCFP. Multivariate logistic regression analysis showed that PFAR was an independent predictor of SCFP (odds ratio: 1.818, *p* = 0.015). Receiver operating characteristic (ROC) curve analysis indicated that PFAR showed a better predictive value of SCFP than fibrinogen or albumin, although not significantly (*p* > 0.05).

**Conclusion:**

PFAR is an independent predictor of SCFP in patients without obstructive CAD. PAFR could improve the predictive value of SFCP than albumin or fibrinogen alone, but not significantly.

## Introduction

Slow coronary flow phenomenon (SCFP) is characterized as slow coronary blood flow in the main vessels without obstructive coronary artery disease (CAD), as determined by the thrombolysis in myocardial infarction (TIMI) frame count (TFC) method via diagnostic coronary angiography [1]. The prevalence of SCFP is 1% to 7% among patients undergoing coronary angiography for suspected CAD [2]. SCFP tends to be considered as a benign phenomenon, however, SCFP has also been associated with life-threatening adverse cardiovascular events such as acute coronary syndrome, ventricular fibrillation, and sudden cardiac death [3]. Previous studies suggested that oxidative stress [4], microvascular [5] and endothelial dysfunction [4, 6], increased resting coronary vasomotor tone [7], platelet function disorder [8], diffuse atherosclerosis [9], systemic/local chronic inflammatory response [4], or combinations of these may have direct and/or indirect roles in the pathophysiology of SCFP. However, the underlying causes remain largely unclear. Therefore, investigating the potential risk factors of SCFP is of vital importance.

As the primary and most abundant plasma protein, albumin is synthesized by the liver and has a key role in the systemic and local inflammatory responses [10, 11]. The plasma albumin level decreases during inflammation. Additionally, as an important antioxidant, plasma albumin effectively reduces free radical injury to the vascular endothelium [12]. Previous studies have suggested that plasma albumin level is inversely correlated with the prevalence, severity, and mortality of CAD [13].

As a precursor of fibrin, plasma fibrinogen is a recognized biomarker of chronic inflammation [14]. In addition to its important role in coagulation, recent studies have indicated a positive correlation with coronary atherosclerosis in stable CAD or STEMI [15, 16]. A recent meta-analysis showed that an increased plasma fibrinogen level is significantly correlated with an increased risk of cardiovascular and all-cause mortality in patients with CAD [17].

Recently, plasma fibrinogen-to-albumin ratio (PFAR) has been suggested as a biomarker of inflammation, which is closely related to a variety of cardiovascular diseases. For example, PFAR has good value in predicting the severity of stable CAD [16]. In addition, the admission PFAR is an independent predictor of no-reflow and short-term mortality in patients with STEMI undergoing primary percutaneous coronary intervention (PCI) [18]. However, there are no data regarding the value of PFAR in the prediction of SCFP among patients without obstructive coronary artery disease. Therefore, we investigated the relationship between PFAR and SCFP in this patient population so as to improve their treatment.

## Methods

### Study population

From January 2021 to January 2023, we consecutively recruited 1085 patients without obstructive CAD according to the diagnostic and exclusion criteria. In total, SCFP was diagnosed in 70 patients. A 1:2 age-matched case–control study was then conducted using unaffected comparators. Ultimately, we included 70 consecutive patients with SCFP and angiographically normal coronary arteries, along with 140 comparators with angiographically normal coronary arteries and normal coronary flow. We excluded patients with acute coronary syndrome (ACS), history of cerebrovascular disease or myocardial infarction, history of revascularization procedures such as PCI or coronary artery bypass grafting, coronary artery aneurysms, coronary spasm or dissection, cardiomyopathy, moderate to severe valvular heart disease, congestive heart failure, non-sinus rhythm, severe liver or renal failure, acute or chronic infection, chronic obstructive pulmonary disease, peripheral vascular disease, autoimmune disease, hematologic disorders, endocrinological disorders (hyper- or hypothyroidism), malignancy, and anemia (hemoglobin level < 12 g/dL for women or < 13 g/dL for men, according to the World Health Organization criteria) [19]. The study protocol was approved by the Ethics Committee of the General Hospital of Ningxia Medical University, and informed consent was obtained from all participants.

### Coronary angiography

The radial artery was the preferred access site for all individuals undergoing coronary angiography. The Judkins technique with 30 frames per second (fps) was used in the procedure. Coronary blood flow was determined by at least two cardiologists using the TFC [1]. For the left anterior descending (LAD) and left circumflex (LCX) arteries, the optimal projection positions for assessing TFC were either the right anterior oblique projection with caudal angulations or the left anterior oblique projection with cranial angulations. The right coronary artery (RCA) was usually assessed in the straight left anterior oblique projection. TFC was calculated as the last frame count minus the initial frame count. The initial frame represented more than 70% contrast agent filling of the lumina of the coronary ostial regions. The last frame was defined as contrast agent appearing at the mustache segment, distal bifurcation segment, and first branch of the posterolateral artery for the LAD, LCX, and RCA, respectively. The corrected TFC (cTFC) for the LAD artery was calculated by dividing the final TFC by 1.7 [20]. The TFCs for normal epicardial coronary arteries were 36.2 ± 2.6 frames for the LAD (21.1 ± 1.5 cTFC for LAD), 22.2 ± 4.1 frames for the LCX, and 20.4 ± 3 frames for the RCA [20]. SCFP was defined as any TFC obtained above these threshold values in at least one of the three coronary arteries. The mean TFC (mTFC) for SCFP or normal coronary flow was determined by adding the TFCs of the LAD, LCX, and RCA, then dividing by three [20].

### Laboratory measurements

After hospitalization, blood samples were obtained before the procedure via the median cubital vein. The samples were then transferred to the central laboratory for testing within one hour after venipuncture. An automatic biochemical analyzer (AU5400; Olympus, Tokyo, Japan) was used to perform the routine biochemical tests. Plasma fibrinogen levels were measured using an automatic coagulation analyzer (STA Compact Max; Stago, Paris, France). PFAR was calculated as plasma fibrinogen concentration divided by albumin concentration and then multiplied by 1000.

### Statistical analysis

IBM SPSS version 20.0 was used for statistical analysis. Normally and non-normally distributed continuous variables are displayed as the mean ± standard deviation and median, respectively. Differences between the two groups were assessed using Student’s t-test and the Mann–Whitney U test. Categorical data are expressed as rates or percentages. The chi-square or Fisher’s exact test was conducted to compare differences. The Pearson or Spearman test was used to assess the correlations between the continuous variables and SCFP. The factors associated with SCFP were determined using univariable regression analysis, and the independent predictors of SCFP were determined using multivariable logistic regression analysis. Receiver operating characteristic (ROC) curve analysis was used to evaluate the accuracy, sensitivity, and specificity of PFAR in distinguishing SCFP with angiographically normal coronary arteries. The Youden index was calculated as sensitivity plus specificity minus one. The predictive value of the factors was determined by the corresponding sensitivity and specificity of the largest number of Youden index. All tests were two-sided, and *p*-values < 0.05 were considered significant.

## Results

### Baseline and clinical characteristics

A total of 210 individuals (70 in the SCFP group and 140 comparators) with angiographically normal coronary arteries were included in this study. Baseline characteristics and medications at the time of hospitalization are shown in Table 1. There were no differences between the SCFP and comparator groups with regard to age, sex, current smoking status, family history of CAD, and diagnoses of dyslipidemia, hypertension, diabetes mellitus, and hyperuricemia (*p* > 0.05). There were no medication differences during hospitalization with regard to angiotensin converting enzyme inhibitors (ACEI), angiotensin II receptor blockers (ARB), angiotensin receptor enkephalinase inhibitors (ARNI), β-antagonists, calcium channel antagonists, antiplatelet agents, statins, and nitrates (*p* > 0.05) (Table 1).

**Table 1:** Baseline characteristics and medication of the two groups

	SCFP group (*n* = 70)	Control group (*n* = 140)	*P* value
Age, years	58.6 ± 10.8	58.6 ± 10.8	1
Male sex, n (%)	47(67.1)	80(57.1)	0.180
Current smoking, n (%)	34(48.6)	73(52.1)	0.662
Family history of CAD, n (%)	13(18.6)	27(19.3)	1
Dyslipidemia, n (%)	14(20.0)	23(16.4)	0.443
Hypertension, n (%)	22(31.4)	41(29.3)	0.752
Diabetes mellitus, n (%)	23(32.9)	44(31.4)	0.876
hyperuricemia, n (%)	12(17.1)	22(15.7)	1
ACEI/ARB/ARNI, n (%)	10(14.3)	18(12.9)	0.830
Beta-blocker, n (%)	11(15.7)	25(17.9)	0.846
Calcium canal blocker, n (%)	10(14.3)	22(15.7)	0.841
Antiplatelet, n (%)	14(20.0)	26(18.6)	0.853
Statin, n (%)	18(25.7)	33(23.6)	0.736
Nitrates, n (%)	19(27.1)	42(30.0)	0.748

*CAD* Coronary artery disease, *ACEI* Angiotensin-converting enzyme inhibitor, *ARB* Angiotensin II receptor blocker, *ARNI* Angiotensin receptor enkephalinase inhibitor

### Laboratory parameters of the two groups

**Fig. 1:**
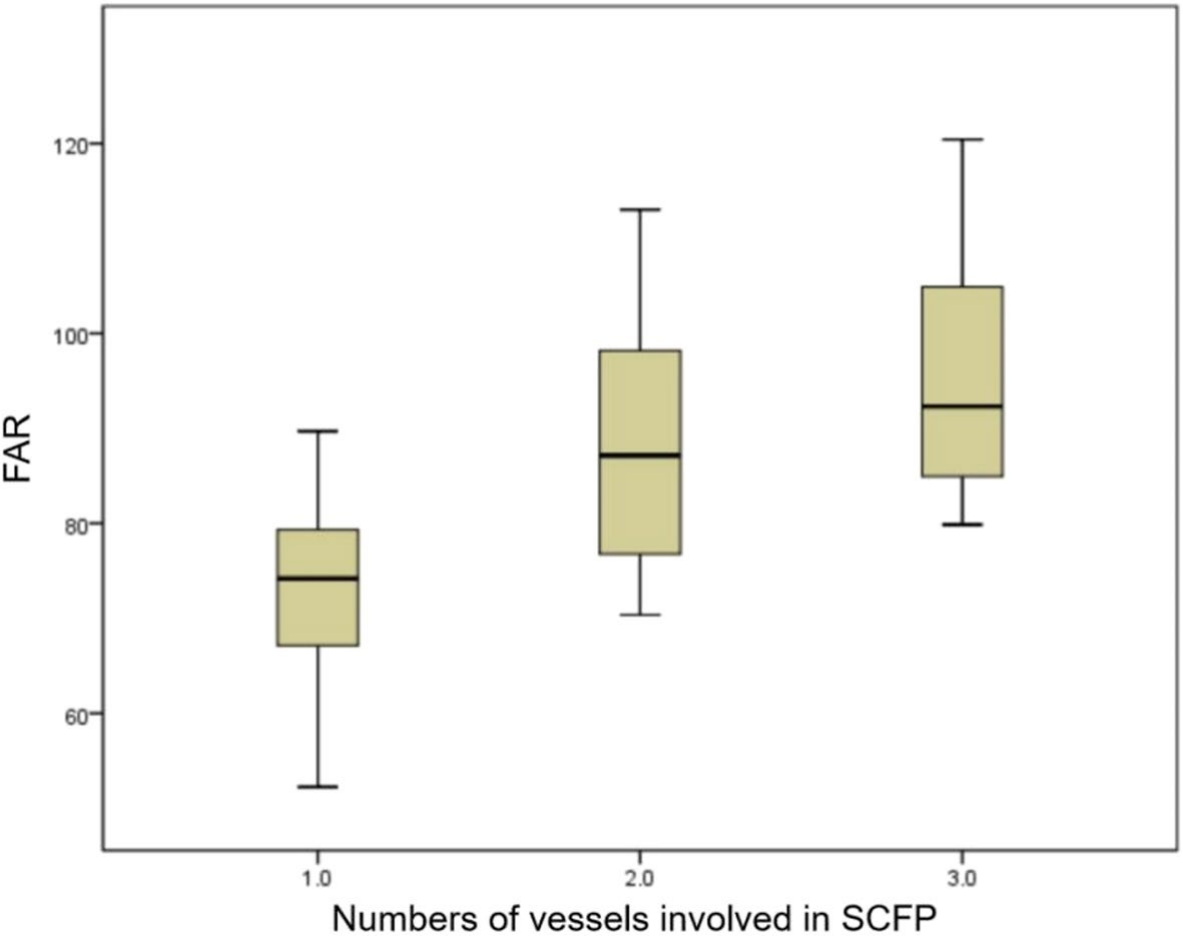
Correlation between the number of vessels involved in SCFP and FAR

The laboratory parameters of the two groups are listed in Table 2. There were no statistically significant differences between the SCFP and comparator groups with regard to lymphocyte and monocyte counts, mean platelet volume (MPV), estimated glomerular filtration rate (eGFR), and plasma concentrations of D-dimer, fasting glucose, triglycerides, high-density lipoprotein cholesterol (HDL-C), and low-density lipoprotein cholesterol (LDL-C) (*p* > 0.05). However, white blood cell count (6.7 ± 1.5 vs 6.3 ± 1.5, *p* = 0.028), neutrophils (4.5 ± 1.2 vs 4.0 ± 1.2, *p* = 0.006), red blood cell distribution width (RDW) (43.0 ± 2.7 vs 42.0 ± 2.3, *p* = 0.011), mean corpuscular volume (MCV) (93.0 ± 6.7 vs 90.0 ± 10.4, *p* = 0.021), platelet distribution width (PDW) (16.1 ± 0.5 vs 15.8 ± 0.7, *p* = 0.001), uric acid level (403.0 ± 87.8 vs 365.3 ± 84.1, *p* = 0.003), fibrinogen level (3.1 ± 0.5 vs 2.9 ± 0.7, *p* = 0.007), PFAR (82.8 ± 15.4 vs 73.1 ± 19.5, *p* < 0.001), total cholesterol level (4.8 ± 1.4 vs 4.0 ± 0.9, *p* < 0.001), and LDL-C (3.2 ± 1.3 vs 2.4 ± 0.8, *p* < 0.001) were higher in the SCFP group than in the comparators. Albumin concentrations were significantly lower in the SCFP group than in the comparators (38.1 ± 2.4 vs 40.2 ± 3.8, *p* < 0.001) (Table 2). Further study showed that PFAR increased with increasing number of vessels involved (Fig. 1).

**Table 2:** Laboratory parameters of the two groups

	SCFP group (*n* = 70)	Control group (*n* = 140)	*P* value
White blood cell, 10^9^/L	6.7 ± 1.5	6.3 ± 1.5	**0.028**
Neutrophils, 10^9^/L	4.5 ± 1.2	4.0 ± 1.2	**0.006**
Lymphocyte, 10^9^/L	1.6 ± 0.4	1.5 ± 0.5	0.493
Monocyte, 10^9^/L	0.4 ± 0.1	0.4 ± 0.1	0.285
RDW, fl	43.0 ± 2.7	42.0 ± 2.3	**0.011**
MCV, fl	93.0 ± 6.7	90.0 ± 10.4	**0.021**
PDW,%	16.1 ± 0.5	15.8 ± 0.7	**0.001**
MPV, fl	9.1 ± 0.8	9.0 ± 1.2	0.651
eGFR, ml/min	82.2 ± 13.5	80.4 ± 15.7	0.440
Uric acid, umol/L	403.0 ± 87.8	365.3 ± 84.1	**0.003**
Albumin, g/L	38.1 ± 2.4	40.2 ± 3.8	** < 0.001**
Fibrinogen, g/L	3.1 ± 0.5	2.9 ± 0.7	**0.007**
D-Dimer, mg/L	0.3 ± 0.2	0.3 ± 0.2	0.128
FAR	82.8 ± 15.4	73.1 ± 19.5	** < 0.001**
Fasting plasma glucose, mmol/l	5.2 ± 0.9	5.2 ± 1.0	0.441
Total cholesterol, mmol/l	4.8 ± 1.4	4.0 ± 0.9	** < 0.001**
Triglyceride, mmol/l	1.5 ± 0.7	1.5 ± 0.9	0.761
HDL-C, mmol/l	1.1 ± 0.3	1.1 ± 0.3	0.634
LDL-C, mmol/l	3.2 ± 1.3	2.4 ± 0.8	** < 0.001**

*RDW* Red blood cell distribution width, *MCV* Mean corpuscular volume, *PDW* Platelet distribution width, *MPV* Mean platelet volume, *eGFR* Estimated glomerular filtration rate, *FAR* Fibrinogen to plasma albumin ratio, *HDL-C* High-density lipoprotein cholesterol, *LDL-C* Low-density lipoprotein cholesterol

### Angiographic characteristics of the two groups

The angiographic characteristics of the included individuals are displayed in Table 3. The TFCs were significantly higher in the SCFP group than in the comparators (*p* < 0.001). In the SCFP group, 58.2% (41/70) of the patients developed SCFP in the LAD artery, 52.9% (37/70) in the LCX, and 68.6% (49/70) in the RCA; 42.9% (30/70) of individuals developed one-vessel SCFP, 32.9% (23/70) developed two-vessel SCFP, and 24.3% (17/70) developed three-vessel SCFP (Table 3).

**Table 3:** Angiographic characteristics of the two groups

	SCFP group (*n* = 70)	Control group (*n* = 140)	*P* value
TIMI frame count			< 0.001
LAD	32.5 ± 15.9	19.4 ± 9.5	
LCX	28.9 ± 10.3	17.6 ± 8.9	
RCA	28.1 ± 9.2	18.1 ± 7.9	
mean TFC	29.8 ± 11.8	18.4 ± 9.2	
Distribution of SCFP			
LAD, n (%)	41(58.2)		
LCX, n (%)	37(52.9)		
RCA, n (%)	49(68.6)		
Numbers of vessels involved in SCFP			
1, n (%)	30(42.9)		
2, n (%)	23(32.9)		
3, n (%)	17(24.3)		

*TIMI* Thrombolysis in myocardial infarction, *LAD* Left anterior descending artery, *LCX* Left circumflex artery, *RCA*: Right coronary artery, *TFC* TIMI frame count, *SCFP* Slow coronary flow fhenomenon

### Predictors of SCFP

**Fig. 2:**
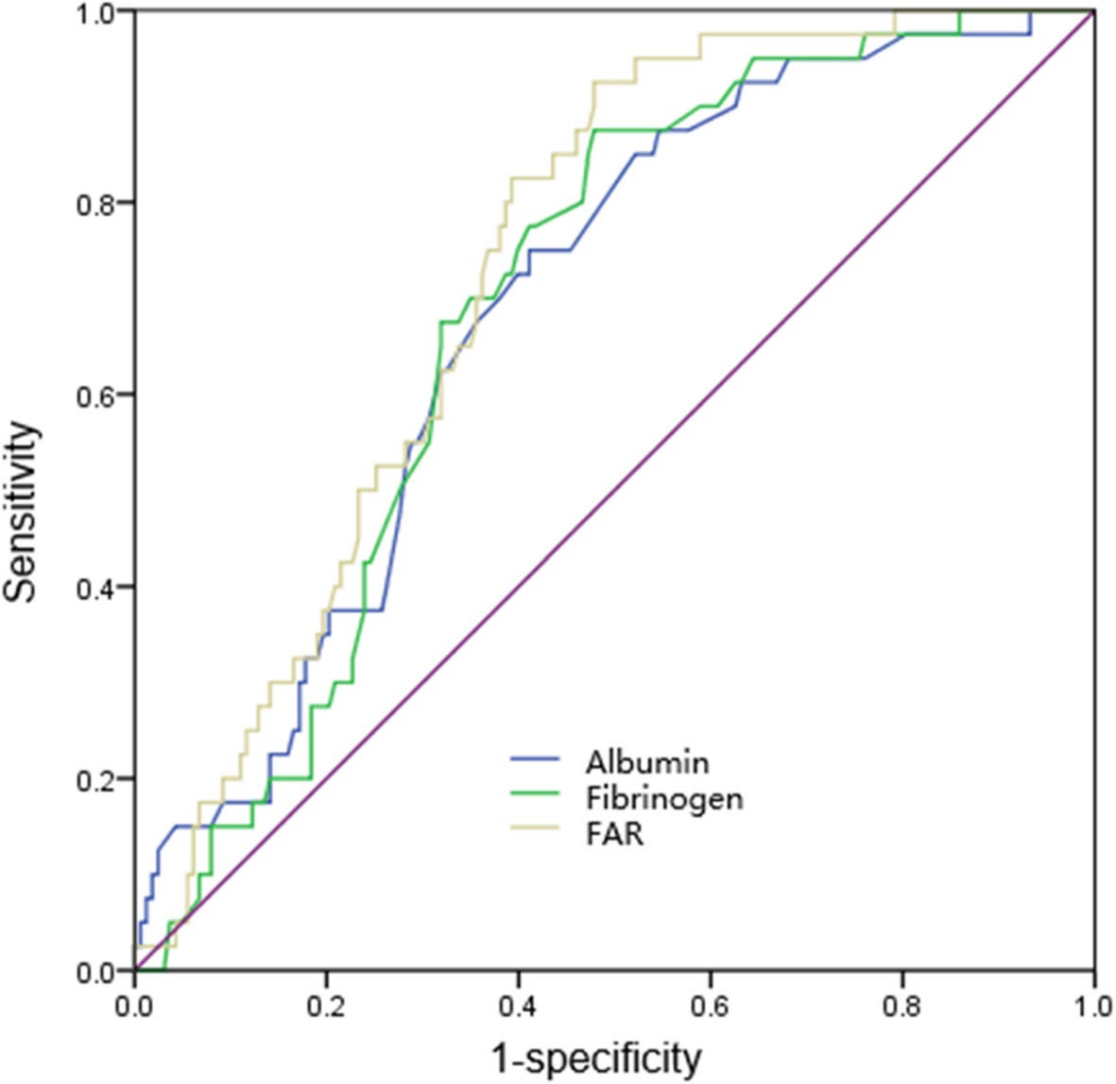
ROC curve showing the predicting value of risk factors for the presence of SCFP

SCFP was associated with female sex, white blood cell count, neutrophil count, RDW, MCV, PDW, PFAR, and levels of uric acid, fibrinogen, albumin, total cholesterol, and LDL-C. After multiple factors were included, fibrinogen levels, albumin levels, and PFAR remained as risk factors for SCFP (Table 4). The ROC curve showed that when PFAR was more than 72.6, the sensitivity and specificity were respectively 80.2% and 72.6%, and the area under the ROC curve (AUC) was 0.731 (95% CI: 0.659–0.803, *p* = 0.028), while when fibrinogen was more than 2.99, the sensitivity and specificity of were 78.3% and 71.2%, and AUC was 0.692 (95% CI: 0.615–0.770, *p* = 0.001) and when albumin was less than 38.6, the sensitivity and specificity of were 77.2% and 70.9%, and AUC was 0.691 (95% CI: 0.609–0.774, *p* = 0.004) (Fig. 2).

**Table 4:** Univariate and multivariate logistic regression analysis for presence of SCFP

	Univariate analysis			Multivariate analysis		
	OR	95% CI	P值	OR	95% CI	P值
Albumin	0.809	0.709–0.929	**0.021**	0.810	0.712–0.932	**0.033**
Fibrinogen	3.562	1.137–12.925	**0.041**	3.509	1.182–13.069	**0.039**
FAR/10	1.826	1.083–5.624	**0.029**	1.818	1.092–6.201	**0.015**

*FAR* Fibrinogen to plasma albumin ratio

## Discussion

In this study, we found that higher fibrinogen level and PFAR, and lower albumin level, were significantly correlated with SCFP. PFAR showed a similarly predictive value for SCFP compared with fibrinogen or albumin. Moreover, the PFAR increased with increasing numbers of vessels involved in SCFP. To the best of our knowledge, this is the first study to investigate the relationship between PFAR and SCFP.

SCFP is characterized as slow blood flow within the main coronary vessels, without obstructive CAD as determined by the TFC method [1]. Previous studies suggested that SCFP usually occurs in young men with smoking history and metabolic syndrome [21]. SCFP is usually considered benign; however, certain studies have revealed associations with myocardial infarction [22], ventricular fibrillation [23], and sudden cardiac death [3]. The exact pathogenesis of SCFP remains unclear. However, oxidative stress [4], microvascular [5] and endothelial dysfunction [4, 6], increased resting coronary vasomotor tone [7], platelet function disorder [8], diffuse atherosclerosis [9], systemic/local chronic inflammatory response [4], or a combination of these may have direct and/or indirect roles in the pathophysiology of SCFP. Traditional cardiovascular risk factors, such as diabetes mellitus, hypertension, dyslipidemia, and hyperuricemia may affect the pathophysiology of SCFP; however, results have varied among different studies. In this study, we observed no correlations between diabetes mellitus, hypertension, hyperuricemia, and SCFP. Although the levels of total cholesterol and LDL-C were significantly higher in the SCFP group than in the comparators, multiple regression analysis showed no correlation. Therefore, we conclude that coronary artery disease and SCFP have quite different risk factors. Establishing a role of traditional cardiovascular risk factors in SCFP requires further investigation. Therefore, examining the potential predictive factors of SCFP is of vital importance to clinical applications.

Inflammation has an important role in the occurrence and development of SCFP. Inflammatory indicators such as C-reactive protein (CRP) [24] and systemic immune-inflammation index [19] are associated with SCFP and have a good predictive value for SCFP. Therefore, we studied commonly used and easily accessible inflammatory biomarkers to investigate the relationship between these factors and SCFP. Fibrinogen is primarily synthesized by the liver and has a key role in the development of inflammation, platelet activation, upregulation of adhesion molecule expression, angiogenesis, and enhancement of macrophage infiltration, all of which aggravate atherosclerotic plaque progression [25]. Increased plasma fibrinogen levels have been observed during the inflammatory state [26], and this can promote platelet aggregation [27]. An elevated plasma fibrinogen level influences coronary blood viscosity, which could introduce endothelial damage and dysfunction of vasoconstriction and vasodilation [28]. Previous studies have suggested a positive correlation between plasma fibrinogen levels and coronary atherosclerosis in stable CAD or STEMI [15, 16]. Lupi et al. [29] discovered that a higher plasma fibrinogen level was a risk factor for in-stent restenosis in patients with ST-elevation myocardial infarction undergoing primary PCI. In this study, we observed significantly higher plasma fibrinogen levels in the SCFP group than in the comparators, and a higher plasma fibrinogen level was an independent predictor of SCFP. We speculate that fibrinogen has an effect on SCFP via local or systemic inflammatory responses, acceleration of coronary atherosclerosis, and endothelial dysfunction.

Albumin is a primary serum protein that regulates colloid osmotic pressure and demonstrates many physiological properties, such as anti-inflammatory, antioxidant, anticoagulant, and antiplatelet activities [30]. The serum albumin level is inversely correlated with local and/or systemic inflammatory status [31]. In addition, a higher plasma albumin level could inhibit platelet activation and aggregation [32], and a decreased albumin level could adversely affect blood viscosity and give rise to endothelial dysfunction [33]. Additionally, as an antioxidant, plasma albumin can capture free radicals, effectively reducing vascular endothelial damage [14]. Previous studies suggested that plasma albumin level was inversely correlated with SYNTAX scores and adverse events in ACS [34]. In a recent study, Kayapinar et al. observed lower levels of plasma albumin in patients with SCFP than in normal comparators [35]. Similar to those results, ours indicated that plasma albumin levels were lower in patients with SCFP. Furthermore, a higher plasma albumin level was an independent predictor of SCFP. We suggest that lower plasma albumin levels could have an important role in the pathogenesis of SCFP by inducing inflammatory reactions, decreasing anti-oxidative stress, and promoting platelet aggregation.

As a new inflammatory biomarker, PFAR integrates fibrinogen and albumin levels into a more comprehensive and sensitive indicator of inflammation, oxidative stress, and the consequent coronary atherosclerosis. Recent studies have suggested that plasma PFAR is closely related to cardiovascular disease. For example, PFAR has been significantly correlated with the severity of coronary stenosis in patients with stable angina [16], adverse outcomes with ACD after PCI [36], in-stent restenosis after PCI [37], and acute kidney injury after PCI [38]. Furthermore, PFAR had a good predictive value in coronary no-reflow phenomenon and short-term prognosis in patients with STEMI undergoing primary PCI [18]. In this study, we therefore evaluated the correlation between PFAR and SCFP, observing a close interrelationship. PFAR increased as the TFC and the numbers of SCFP-affected vessels increased. A higher plasma PFAR was an independent predictor of SCFP. The mechanisms associating PFAR with SCFP are as follows: (1) local or systemic inflammatory response as a possible first and main mechanism; (2) oxidative stress and endothelial dysfunction; and (3) coronary or systemic atherosclerosis relating to various cardiovascular risk factors, inflammatory responses, and endothelial dysfunction.

SCFP is a complex disease with many unknown risk factors. In this study, we combined albumin and fibrinogen levels to better reflect inflammatory and oxidative stress status. We conclude that PFAR could improve the prediction of SCFP compared to albumin or fibrinogen levels alone, although not substantially. As an easily acquired indicator that is widely used in clinical practice, we believe that the PFAR has an important and promising role in the evaluation of SCFP in patients without obstructive CAD. However, this study also had certain limitations. First, this was a single-center study with a small sample size, which can lead to selection bias. Second, although multivariate analyses were conducted, residual covariates may still exist, and this may affect the predictive value of PFAR. Third, we were unable to include all inflammatory indicators, such as C-reactive protein level. Finally, the patients included in this study represented a specific population; large-sample, multi-center studies are needed to validate our conclusions.

## Conclusion

PFAR independently predicts SCFP in patients without obstructive CAD. PFAR could improve the prediction of SCFP more than albumin or fibrinogen levels alone, although not substantially.

## Data Availability

The datasets generated and analysed during the current study are not publicly available due to a further study of this area but are available from the corresponding author on reasonable request.

## Abbreviations

SCFP: Slow coronary flow phenomenon
PFAR: Plasma fibrinogen to albumin ratio
CAD: Coronary artery disease
TIMI: Thrombolysis in Myocardial Infarction
TFC: TIMI frame count
LAD: Left anterior descending
LCX: Left circumflex
RCA: Right coronary artery
ACEI: Angiotensin converting enzyme inhibitors
ARB: Angiotensin II receptor blocker
ARNI: Angiotensin receptor enkephalinase inhibitor
MPV: Mean platelet volume
eGFR: Estimated glomerular filtration rate
HDL-C: High-density lipoprotein cholesterol
LDL-C: Low-density lipoprotein cholesterol
RDW: Red blood cell distribution width
MCV: Mean corpuscular volume
PDW: Platelet distribution width
CRP: C-reactive protein
